# Vaccines as Global Health Security Infrastructure: Insights from a Descriptive Analysis of Vaccines Europe Members’ Clinical Pipelines

**DOI:** 10.3390/vaccines14050456

**Published:** 2026-05-19

**Authors:** Charlotte Vernhes, Kateryna Khmilevska, Alexis Caron, Emanuele Ciglia, Rosybel Drury, Judith Perez-Gomez, Volker Vetter

**Affiliations:** 1 Vaccines Europe, European Federation of Pharmaceutical Industries and Associations (EFPIA), 1000 Brussels, Belgium; 2 Sanofi, 94250 Gentilly, France; 3 Seqirus, 53035 Monteriggioni, Italy; 4 Merck Sharp & Dohme (MSD), 69002 Lyon, France; 5 Valneva, 69002 Lyon, France; 6 GlaxoSmithKline (GSK), 1300 Wavre, Belgium

**Keywords:** infectious diseases, vaccines, prophylactic mAbs, pipeline, immunisation, innovation, public health, horizon scanning, emerging pathogens, AMR

## Abstract

**Background/Objectives:** Vaccine development pipelines are forward-looking indicators of public health preparedness, reflecting the capacity to address unmet medical needs and emerging threats. This descriptive analysis aims to characterise the 2025 clinical-stage pipeline of infectious disease vaccines and prophylactic monoclonal antibody candidates developed by Vaccines Europe member companies, and to describe how pipeline characteristics address evolving public health priorities. **Methods:** A descriptive analysis was conducted using publicly available data compiled in the Vaccines Europe Pipeline Review 2025, with validation by participating companies. Candidates in clinical development or regulatory review were classified using a standardised framework by pathogen/disease, target population, public health priority, and technologies. **Results:** The Vaccines Europe member company pipeline comprises 91 candidates across clinical development phases, 19% of which are in Phase III and 7% undergoing regulatory review. This pipeline is predominantly targeting respiratory pathogens (75%), with a strong life-course focus (85% evaluated in adults and/or older adults), and sustained activity in bacterial pathogens relevant to antimicrobial resistance. Notably, 41% of candidates were classified as addressing diseases, disease combinations, or indications for which no licenced preventive product exists. This category includes candidates targeting diseases without a preventive solution, as well as novel combination vaccines and therapeutic approaches in areas where individual components or preventive vaccines are already available. This captures vaccines candidates in different stages of development, not necessarily first-in-disease innovation. The pipeline shows broad technological diversity (12 technologies), dominated by RNA approaches and multivalent candidates, with growing focus on climate-sensitive, zoonotic, and pandemic-prone pathogens. **Conclusions:** Within the pipeline of Vaccines Europe member companies, this analysis describes development activity oriented toward broader prevention, platform-based approaches, and preparedness-relevant targets. As a structured and recurring annual assessment, the Vaccines Europe Pipeline Review supports horizon scanning and evidence-based dialogue between industry and vaccine ecosystem stakeholders. In order to maximise the impact of vaccine development pipelines to public health, predictable investment, streamlined trial and regulatory pathways, strong surveillance, and real-world data systems, coordinated decision-making is required to enable timely and equitable access, and complementary incentive and procurement reforms.

## 1. Introduction

### 1.1. The Transformative Impact of Fifty Years of Vaccine Innovation

Vaccination has fundamentally improved global health and social stability over the last century by preventing infectious diseases, reducing long-term complications, and supporting economic and social systems. According to the World Health Organisation (WHO), routine vaccination programmes targeting 14 diseases have saved an estimated 154 million lives in the past 50 years, representing 40% of the global improvement in infant survival, with the measles vaccine alone accounting for 60% of these lives saved [1]. In recent years, new respiratory syncytial virus (RSV) vaccines and monoclonal antibodies (mAbs) [2,3] have significantly reduced paediatric hospitalisations and healthcare strain. These achievements underscore immunisation as a cornerstone of both public health and broader societal resilience.

Over the past decades, the impact of vaccination has been expanding beyond childhood diseases. The human papillomavirus (HPV) vaccine can reduce cervical cancer risk by over 80% [4] when given in early adolescence. Together with screening, new HPV vaccine products protecting against infection by all high-risk HPV variants offer a real prospect of eliminating HPV-related cancers. The COVID-19 pandemic demonstrated that adult immunisation at scale is both feasible and effective, achieving high coverage among healthcare workers and older adults worldwide [5,6].

Additionally, there is growing evidence of the impact of infection prevention on downstream complications, including cardiovascular [7], respiratory [8], oncologic [9], neurodegenerative [10], and autoimmune diseases [11]. These broader effects are being explored in ongoing research, and while they currently do not constitute established vaccine indications, they could be considered for their potential longer-term public health implications.

### 1.2. Evolving Infectious Disease Threats and the Expanding Value of Prevention

Demographic and epidemiological shifts are reshaping infectious disease risk and driving the evolution of immunisation programmes. Globally, older adults now outnumber children under five, with those aged ≥60 years projected to reach over 2 billion by 2050 [12]; in the European Union, the median age has already reached 44.7 years [13]. Ageing populations face increased susceptibility to infections, higher rates of hospitalisation and long-term disability, and elevated risks of downstream non-communicable sequelae. These demographic shifts increase the importance of adult immunisation programmes, yet uptake of routine adult vaccines (e.g., against influenza and pneumococcal disease) remains suboptimal, reflecting immunisation systems that are still largely structured around paediatric delivery and therefore leaving a substantial burden of vaccine-preventable morbidity and mortality.

Alongside demographic change, the acceleration of microbial evolution is contributing to the growing burden of antimicrobial resistance (AMR), which is associated with rising mortality, escalating healthcare costs, and deepening socioeconomic inequalities; it also threatens modern healthcare by reducing the effectiveness of antibiotics needed for routine surgery, cancer treatment, neonatal care, and the management of immunocompromised patients. Vaccination is a critical prevention strategy that complements antimicrobial stewardship and infection prevention and control by reducing the incidence of infections that lead to antibiotic prescribing. It contributes through direct effects, preventing bacterial disease and transmission, including infections caused by resistant strains, and indirect effects, reducing inappropriate antibiotic use for viral respiratory illnesses and lowering the risk of secondary bacterial infections following viral disease [14]. Recent WHO estimates suggest that existing vaccines could avert up to 515,000 deaths annually, prevent 2.5 billion antibiotic doses, and save up to USD 30 billion in hospital costs [15], underscoring immunisation as a scalable upstream intervention to mitigate AMR while strengthening health system resilience.

In parallel, climate change is altering infectious disease dynamics and driving the need for adapted prevention strategies. Rising temperatures and changing environmental conditions are expanding the geographic range of vectors such as ticks and mosquitoes, increasing transmission of diseases including Lyme disease, West Nile virus, dengue, chikungunya, malaria, and yellow fever [16,17]. Climate-related disruption of food and water systems heightens the risk of water- and food-borne infections, while displacement and urban crowding amplify transmission in vulnerable populations. Meanwhile, zoonotic pathogens, responsible for around 60% of human infectious diseases and causing an estimated 2.7 million deaths and 2.5 billion [18] illnesses, continue to pose significant pandemic risks, as illustrated by COVID-19 and the resurgence of mpox [19].

In response to shifting demographics, evolving epidemiology, and the continued development of new vaccines and technologies, many European countries are reviewing and expanding their routine immunisation schedules across age groups [20,21]. This includes the introduction of new vaccines, extension of existing indications, and adjustments to schedules in line with evolving epidemiology and emerging evidence. These changes reflect a gradual shift towards more comprehensive immunisation strategies that better align with changing disease patterns and population needs.

Together, these trends reinforce that immunisation, encompassing both active (vaccines) and passive (prophylactic mAbs) approaches, is not only a tool for infectious disease control, but a strategic investment in long-term population health, health system sustainability, and social and economic resilience [22]. By preventing illness, hospitalisation, and long-term complications across the life course, vaccines protect healthcare capacity, reduce productivity losses, and help mitigate the economic disruption associated with outbreaks and pandemics. Evidence from ten high-income countries suggests that adult immunisation programmes (influenza, pneumococcal disease, herpes zoster, and RSV) can yield up to a 19-fold return on investment when broader benefits, such as productivity gains and caregiver burden reduction, are included [23]. While these estimates depend on underlying assumptions and may not be directly generalisable across settings, they illustrate the potential scale of societal value of adult immunisation. Ensuring that prevention is adequately prioritised in budget decisions and embedding life-course immunisation as a core component of health systems is therefore essential to address evolving demographic and environmental risks, strengthen preparedness, and maximise the full health and societal value of vaccination.

### 1.3. Innovation Pathways: Breakthrough Discoveries and Incremental Progress

Vaccine innovation has historically progressed through a combination of breakthrough scientific discoveries and sustained incremental advances, both of which are essential in addressing evolving infectious disease threats and their long-term impact. Transformative breakthroughs often stem from fundamental insights in immunology, structural biology, or pathogen biology that unlock previously inaccessible targets or mechanisms of protection. One example is the elucidation of the prefusion conformation of the RSV F protein [24], which enabled the rational design of stabilised antigens capable of eliciting potent neutralising immune response while avoiding potential adverse reactions due to antibody-dependent enhancement [25]. This led to the licensure of multiple RSV vaccines and monoclonal antibodies for use across the life-course and informed antigen design principles applied to other viral vaccines, including during the COVID-19 pandemic.

Concurrently, genome-based antigen-discovery approaches such as reverse vaccinology have broadened the range of bacterial pathogens that can be targeted through vaccination, including those characterised by high antigenic variability and advanced immune evasion mechanisms, which are highly relevant in the context of AMR. At the same time, incremental innovation in antigen composition, formulation, adjuvants, delivery platforms, manufacturing, and immunisation strategies, illustrated by multivalent and combination vaccines, has cumulatively expanded vaccine effectiveness, programmatic efficiency, and population coverage. These breakthrough and incremental pathways are complementary, jointly shaping how scientific advances are translated into vaccines that address diverse and changing public health needs. They also highlight the importance of sustained investment and predictable innovation ecosystems to ensure preparedness for both known and emerging infectious disease threats.

### 1.4. Horizon Scanning and the Role of Vaccine Pipelines

Vaccine development pipelines are forward-looking indicators of public health preparedness, with their composition, maturity, and technological diversity reflecting a health system’s capacity to anticipate emerging threats and reduce unmet infectious disease burdens. Systematic monitoring of those pipelines enables earlier alignment between innovation trajectories and public health planning, helping to anticipate gaps, prioritise investments, and coordinate surveillance, regulatory, and manufacturing preparedness before threats fully materialise or new interventions become available.

Combined with global frameworks led by the WHO, such as the 2024 Bacterial Priority Pathogens List [26] and the list of priority endemic pathogens, pipeline assessments can help guide EU research funding (e.g., Horizon Europe, the Health Emergency Preparedness and Response Authority (HERA)) towards remaining areas of high unmet medical need, including vaccines targeting antibiotic-resistant bacteria, high-burden endemic diseases (e.g., human immunodeficiency virus (HIV), tuberculosis, and malaria), and pathogens with epidemic or pandemic potential (including coronaviruses, influenza viruses, dengue, Nipah, Lassa, Ebola, and “Disease X”). In this sense, horizon scanning shifts vaccine portfolios from reactive responses to proactive preparedness strategies.

Vaccines Europe (VE), the vaccine-specialised group of the European Federation of Pharmaceutical Industries and Associations (EFPIA), represents vaccine companies operating in Europe [27]. In 2022, VE launched its Pipeline Review (referred to as “pipeline” throughout the manuscript), an annual analysis of clinical-stage vaccine and prophylactic mAb candidates targeting infectious diseases in its members’ pipelines [28]. While these reviews do not cover the entire vaccine industry, they systematically track and present a snapshot of vaccine innovation among VE member companies. To our knowledge, this is the first structured, longitudinal review of clinical-stage infectious disease vaccine and prophylactic monoclonal antibody candidates across multiple vaccine companies operating in Europe. The objective is to support horizon scanning and early dialogue between the vaccine industry and health authorities by aggregating evidence on technologies, targets, stages, and public-health priorities, thereby informing value assessment, immunisation financing, and national preparedness.

## 2. Materials and Methods

### 2.1. Data Source and Scope

This article is based on publicly available data analysed and compiled in the VE Pipeline Review 2025 edition [29]. In its fourth edition, published on 1 December 2025, the pipeline provides updated data collected and validated through 31 August 2025, from VE’s 16 member companies at the time (Abbott, AstraZeneca, Bilthoven Biologicals, CSL Seqirus, CureVac, GSK, HIPRA, Johnson & Johnson, Moderna, MSD, Novavax, Pfizer, Sanofi, Takeda, Valneva, and Vaxcyte).

The scope includes vaccines and prophylactic mAbs candidates (referred to as “candidates” throughout the manuscript) addressing infectious diseases and their consequences. Candidates against non-infectious targets (e.g., cancer) are considered out of scope.

This review focuses on candidates in clinical development (Phase I–III) as well as those under regulatory review at the time of data collection. Candidates in preclinical development and authorised products were excluded. The highest global development status was considered for the analysis (e.g., candidates in Phase I/II clinical trials were counted as Phase II). Furthermore, candidates under active review by any Regulatory Authority (not necessarily in Europe) were counted as under ‘Regulatory Review’, and products that have received marketing authorisation in any region in the world are no longer included in the VE Pipeline Review.

### 2.2. Data Collection and Classification

Pipeline candidates were classified using a structured framework defined prior to data collection and applied consistently across all candidates to enable consistent aggregation and interpretation of clinical-stage innovation across company pipelines. Classification was applied across five main dimensions:

1. Classification by target population. Candidates were categorised according to the population evaluated in the clinical development programme, as described in public sources. Target population categories were assigned based on the trial population rather than by applying mutually exclusive biological cut-offs. Each candidate was assigned to one primary population category: paediatric, adults, older adults, paediatric + adults, and adults + older adults. Where maternal immunisation was evaluated, candidates were captured within the adult category and described separately.
2. Classification by pathogen/disease area. Each candidate was categorised according to its pathogen target or disease indication, enabling analysis by infectious disease area and comparison across pipeline segments (e.g., routine immunisation [21], transmission route). Candidates addressing multiple pathogens or diseases (i.e., combination vaccines) were captured under each relevant section.
3. Classification by nature of innovation. The pipeline is divided into two broad categories:
  a. “New targets”, a category including candidates targeting diseases, combinations of diseases, or infectious syndromes, for which no vaccine or prophylactic mAbs product exists globally. This category should not be interpreted as first-in-disease, as it includes candidates targeting infectious diseases without any registered vaccine or prophylactic mAb product, novel combination vaccine candidates for when there is no registered vaccine or prophylactic mAb product covering all target pathogens or diseases, and therapeutic vaccine candidates in areas where only preventive vaccines or prophylactic mAb products are available.
  b. “Further development”, a category including candidates aiming at further developing existing vaccines or finding new approaches to address a disease. This includes developments such as improving formulations, expanding vaccine use to new populations, and developing multivalent or combination vaccines for targets where there is already at least one approved product on the market covering the same strains or pathogens.
4. Classification by public health challenge addressed. To support horizon scanning and policy relevance, candidates were additionally labelled according to the public health challenges they address, such as AMR, climate change-related threats including vector- and water-borne diseases, and emerging infectious diseases covering zoonoses and pandemic preparedness. Standardised labels informed by previously established frameworks (e.g., WHO bacterial priority pathogens list 2024 [26]), global health threat assessments (e.g., Global Alliance for Vaccines and Immunisation (GAVI) [30]), and other recognised sources [18,31] were used descriptively to contextualise pipeline activity rather than to formally assess alignment against predefined benchmarks or criteria. Candidates belonging to several categories were captured under each relevant section to support horizon scanning.
5. Classification by technology. Candidates were classified by immunisation technology to analyse innovation trends and the diversity of scientific approaches represented in clinical pipelines.

This analysis provides an anonymised, aggregated overview of candidates in the clinical pipelines of the companies listed above. The data included in the annual publication were collected from public sources: company websites (i.e., innovation pipeline pages consulted until August 2025), quarterly reports, and the intelligence platforms Citeline Pharmaprojects [32], and Citeline Trialtrove [33].

Once compiled, all data were individually verified by each company prior to aggregation and publication to ensure it was accurate and most up to date at the time of collection (i.e., 31 August 2025). Company input was limited to verification of factual data (e.g., development stage, target pathogen), while classification and aggregation were conducted centrally by the VE secretariat without the involvement of individual member companies and using predefined criteria.

### 2.3. Longitudinal Comparison

To assess the evolution of the clinical vaccine pipeline over time (2022–2025), a structured longitudinal analysis was conducted using publicly available data analysed and compiled in the VE Pipeline Review 2022, 2023, 2024, and 2025 editions [29]. Four predefined annual metrics were considered: attrition rate, registration rate, progression rate, and pipeline entry rate. These indicators were calculated for each year from 2023 to 2025, assessing change from the previous year (i.e., 2022–2023, 2023–2024, and 2024–2025), to describe pipeline dynamics.

The annual attrition rate was defined as the percentage of candidates discontinued during a given annual period relative to the previous year’s pipeline. Discontinuations included candidates formally terminated at any stage of clinical development. Note that this does not include candidates removed from the pipeline because the company was no longer a member of VE.

The annual registration rate was defined as the percentage of candidates that received marketing approval during a given annual period relative to the previous year’s pipeline. Notably, candidates receiving approval from any regulatory authority globally (including non-EU) were considered registered.

The annual progression rate was defined as the percentage of candidates that advanced to a higher stage of development (e.g., Phase I to Phase II, Phase II to Phase III, or Phase III to regulatory review) during a given annual period relative to the previous year’s pipeline. For candidates spanning combined phases (e.g., Phase I/II), advancement to the next-highest distinct phase was considered progression.

The annual pipeline entry rate was defined as the percentage of new candidates entering clinical development (Phase I or higher) during a given annual period relative to the previous year’s pipeline.

## 3. Results

### 3.1. Overview of the Clinical-Stage Pipeline of Vaccines Europe Member Companies

Overall, this analysis identified a total of 91 candidates, comprising 95% (86) prophylactic and 2% (2) therapeutic vaccines, and 3% (3) prophylactic mAbs, all targeting infectious agents.

**Figure 1:**
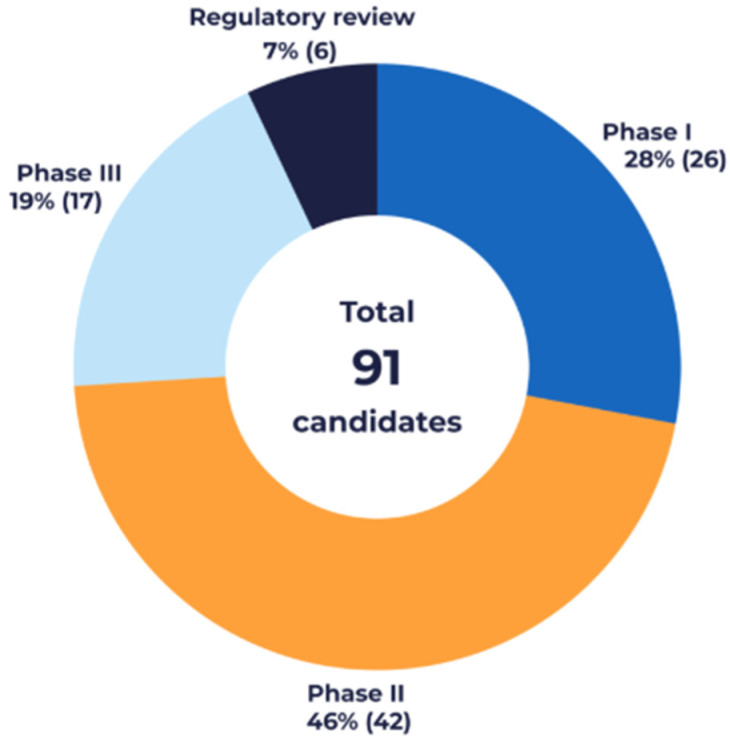
Vaccines Europe members’ 2025 pipeline at a glance.

Of those, 28% (26) candidates were in Phase I, 46% (42) in Phase II, and 19% (17) in Phase III of clinical development (Figure 1). Additionally, 7% (six) were undergoing regulatory review globally in 2025.

**Figure 2:**
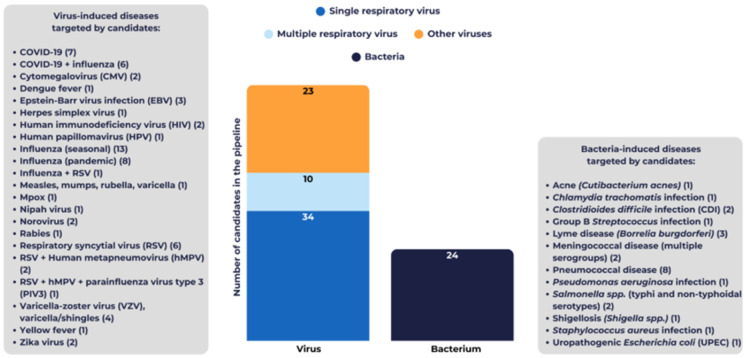
Target microorganisms in Vaccines Europe members’ 2025 pipeline.

While most candidates are aimed at combating viral infectious diseases—74% (67), a substantial portion focus on bacterial infectious diseases—26% (24) (Figure 2). In this analysis, there were no candidates targeting fungal or protozoan pathogens.

**Figure 3:**
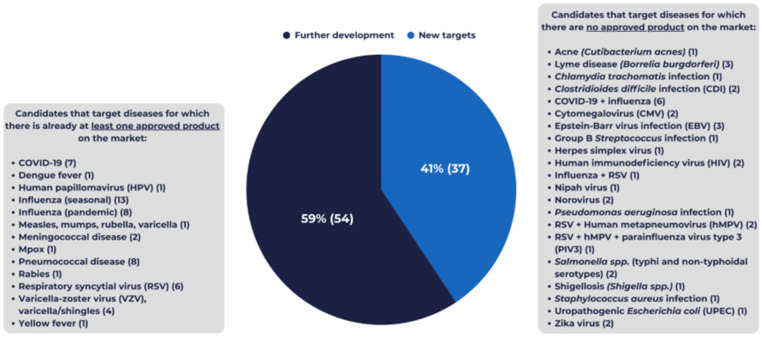
Candidates within Vaccines Europe members’ pipeline targeting diseases for which there is no registered vaccine or prophylactic mAb (“New targets”, light blue) vs. those further developing existing products (“Further development”, dark blue). Note: The “New targets” category includes candidates targeting: infectious diseases without any registered vaccine or prophylactic mAb product; combination candidates where vaccine or prophylactic mAb products are licenced for individual pathogens, but not for the specific combination (e.g., COVID-19 + seasonal influenza); and therapeutic candidates where only a preventive vaccine or mAb product was licenced (none in the 2025 pipeline). It should not be interpreted as synonymous with first-in-disease innovation.

As illustrated in Figure 3, 41% of the candidates (37) are categorised as aiming to address “New targets”. A majority of candidates (27) target infectious diseases or pathogens without any registered vaccine or prophylactic mAb product globally: acne (1), Lyme disease (3), *Chlamydia trachomatis* (1), *Clostridioides difficile* (2), cytomegalovirus (CMV) (2), Epstein–Barr virus (EBV) (3), group B *Streptococcus* (GBS) (1), herpes simplex virus (HSV) (1), HIV (2), Nipah virus (1), norovirus (2), *Pseudomonas aeruginosa* (1), *Salmonella* spp. (typhi and non-typhoidal) (2), *Shigella* spp. (1), *Staphylococcus aureus* (1), Uropathogenic *Escherichia coli* (UPEC) (1), and Zika virus (2). The rest (10) are novel combination vaccine candidates (i.e., no registered vaccine or prophylactic mAb product covering all target pathogens): COVID-19 and influenza, influenza and RSV, RSV and human metapneumovirus (hMPV), RSV and hMPV, and parainfluenza virus type 3 (PIV3). Notably, there are no standalone hMPV or PIV3 registered vaccine products available to date.

In contrast, 59% (54) of the pipeline is categorised as “further development”, addressing pathogens or diseases for which registered vaccine or prophylactic mAb products already exist, with the aim of broadening the spectrum of interventions available to health professionals and populations offered vaccination.

### 3.2. Public Health Priorities Reflected in the 2025 Vaccines Europe Members’ Pipeline

**Figure 4:**
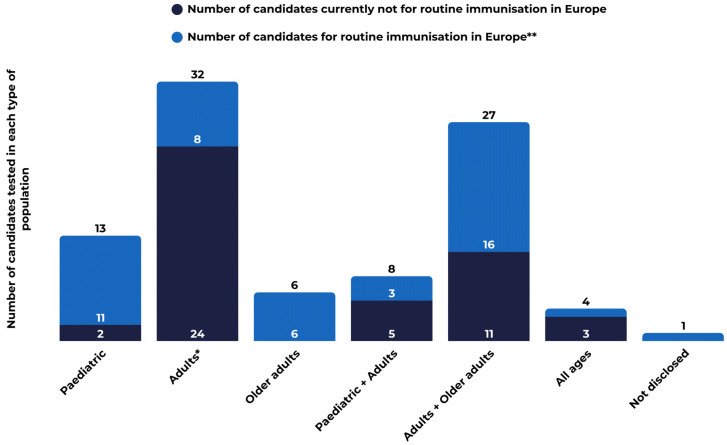
Life-course immunisation: target populations in Vaccines Europe members’ pipeline. * Includes one candidate for maternal immunisation. ** Includes combination candidates for seasonal influenza and RSV.

The clinical pipeline reflects a clear life-course immunisation orientation. Most candidates, 85% (77), are evaluated in adult and/or older-adult populations, which is consistent with expanding prevention beyond childhood programmes and addressing the disproportionate burden of severe disease in older adults and individuals with comorbidities (Figure 4). Maternal immunisation is represented in the 2025 portfolio, with one vaccine candidate targeting GBS, in a Phase III clinical trial.

Approximately 51% (46) of the candidates in development are intended for routine immunisation in Europe. Several candidates are being evaluated in both paediatric and adult populations (Figure 4). Targeted pathogens include seasonal influenza virus (20), RSV (10), varicella-zoster virus (4), HPV (1), measles, mumps, rubella, and varicella virus (MMRV combination vaccine) (1), as well as pneumococcal (8) and meningococcal (2) pathogens.

In 2025, among the 91 candidates, 29% (26) target infectious diseases transmitted through infectious body fluids, 17% (15) through direct contact (physical and surface contact), 8% (7) through vectors, and approximately 7% were associated with sexually transmitted (6) and food-/water-borne infections (6). These categories are not mutually exclusive, as some pathogens fall under multiple transmission routes.

**Figure 5:**
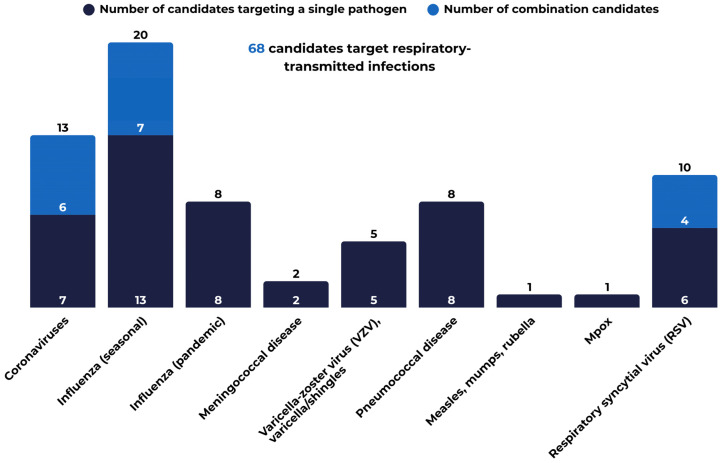
Monovalent and combination vaccine candidates against respiratory infectious diseases in Vaccines Europe members’ pipeline.

Candidate vaccines to prevent or treat respiratory infections constitute the largest proportion, accounting for 75% (68) of all candidates. As shown in Figure 5, this group includes candidate vaccines targeting the influenza virus (seasonal and pandemic), RSV, coronaviruses, and bacterial respiratory diseases. Development efforts also include combination vaccines targeting multiple respiratory viruses simultaneously, reflecting a shift toward broader prevention of respiratory illnesses rather than single-pathogen approaches. Ten such combination vaccines are currently in development (Figure 5), including six COVID-19 + seasonal influenza candidates, two RSV + hMPV candidates, one RSV + hMPV + PIV3 candidate, and one seasonal influenza + RSV candidate.

**Figure 6:**
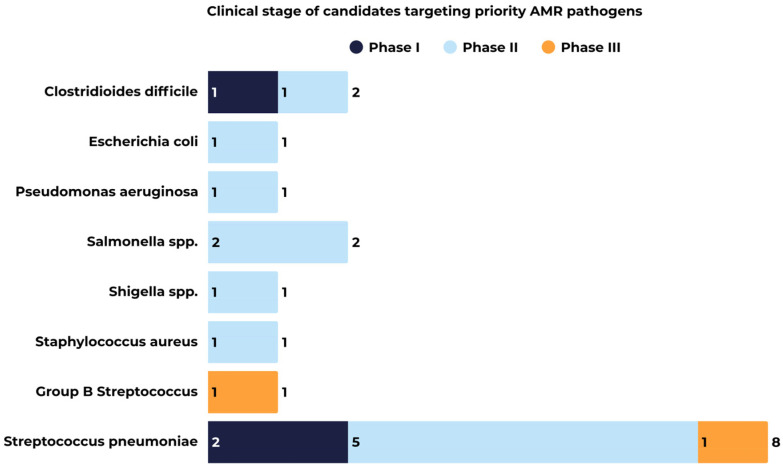
AMR-relevant bacterial candidates in the Vaccines Europe members’ pipeline.

The 2025 pipeline included 17 candidates targeting eight bacterial pathogens associated with significant AMR, with candidates distributed among all clinical phases (Figure 6). These targets align with international priorities, as seven of the eight pathogens are included in the WHO 2024 Bacterial Priority Pathogens list [26], with one listed as critical (i.e., *Escherichia coli*), four as high (i.e., *Pseudomonas aeruginosa*, *Salmonella* spp., *Shigella* spp., *Staphylococcus aureus*), and two as medium (i.e., Group B *Streptococcus*, *Streptococcus pneumoniae*). This distribution underscores the role of vaccination as a preventive strategy aimed at reducing infectious diseases and, consequently, the need for antimicrobial treatment.

**Figure 7:**
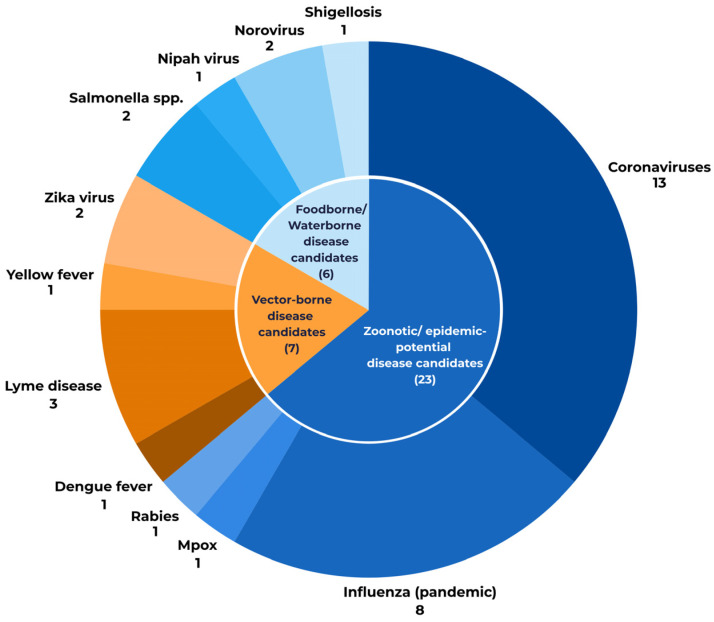
Number of candidates in Vaccines Europe members’ pipeline targeting climate-sensitive and zoonotic threats grouped according to infectious disease mode of transmission.

Vaccine innovation is also responding to shifting disease ecology and environmental changes. The pipeline includes candidates targeting climate-sensitive risks, specifically vector-borne and food-borne/water-borne pathogens (Figure 7). Many of these candidates, including those against dengue and yellow fever, are being developed to address substantial and longstanding infectious disease burdens in endemic regions, while also recognising that climate change, global travel, migration, and expanding vector ranges are extending these risks to new, previously non-endemic populations.

The clinical pipeline of vaccines and mAb candidates includes a significant focus on emerging threats, with 34% (31) of candidates targeting diseases with zoonotic or pandemic potential. VE members are addressing the challenge of zoonotic diseases by researching vaccines against COVID-19, dengue fever, pandemic influenza, Lyme disease, rabies, Nipah virus disease, salmonellosis, and yellow fever (Figure 7). These candidates are critical components of EU horizon scanning, serving as indicators for future public health countermeasures and for building rapid-response capabilities.

### 3.3. Technological Innovation Strategies

**Figure 8:**
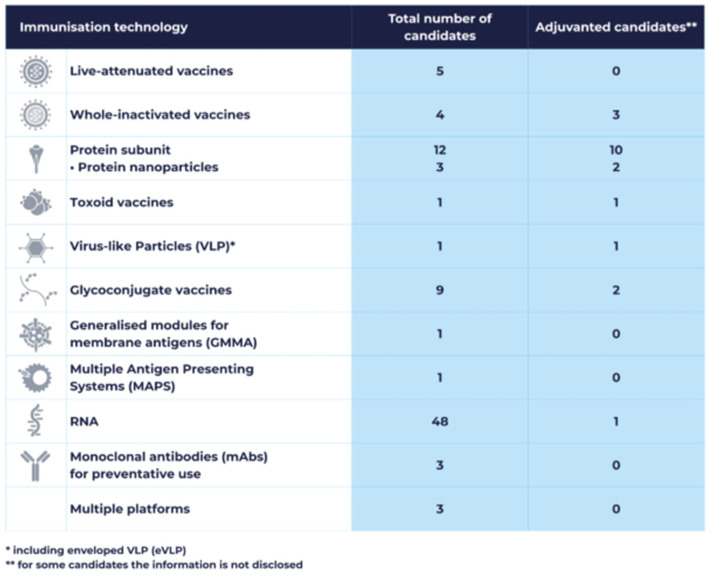
Candidate vaccines classified by technology type (adjuvantation indicated) in the Vaccines Europe members’ 2025 pipeline.

In 2025, candidates in the VE members’ clinical pipeline were based on 12 distinct vaccine technologies (Figure 8), reflecting the diversity of scientific approaches represented at the clinical stage.

RNA-based vaccine candidates constituted the largest technology category, with 48 candidates (53% of the pipeline), primarily targeting infectious diseases caused by viral pathogens, with a smaller number directed against bacterial diseases. Protein subunit vaccines accounted for 12 candidates (13%), including three nanoparticle-based formulations. Nine candidates were glycoconjugates (10%), five were live-attenuated vaccines (5%), and four were whole-inactivated vaccine candidates (4%). Toxoids, Generalised Modules for Membrane Antigens (GMMA), Multiple Antigen Presenting Systems (MAPS), and Virus-like Particles (VLPs) were each represented by one candidate. Three candidates were based on combinations of multiple technologies. Finally, three candidates were prophylactic monoclonal antibodies.

Many vaccine candidates include adjuvants to enhance the immune response. These range from established adjuvants to company-developed innovative formulations and may contain natural or synthetic substances such as oils, bacterial lipids, salts, surfactants, saponins, liposomes, and proteins.

**Figure 9:**
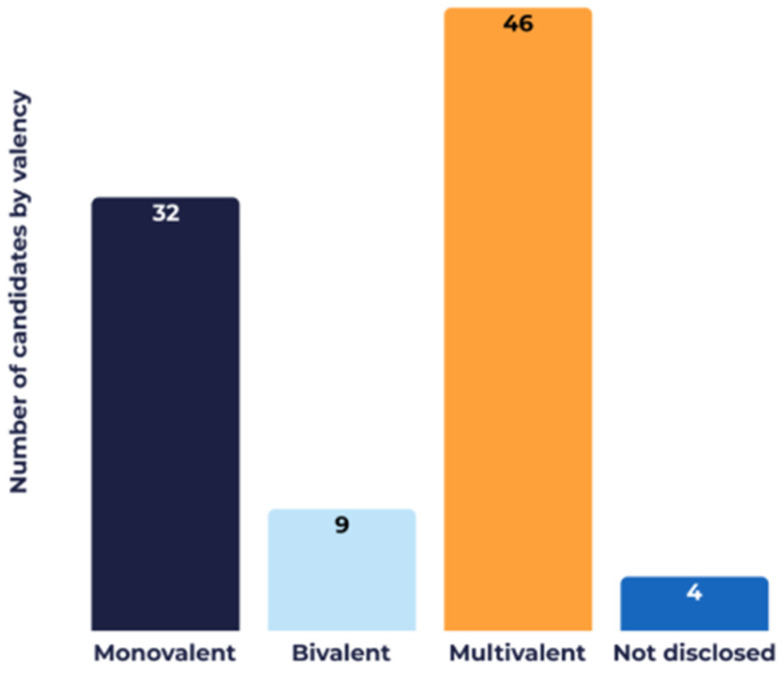
Valency (number of strains of the same pathogen) of the candidates in development in the Vaccines Europe members’ pipeline.

Some of these technologies enable the development of multivalent vaccines (targeting multiple strains of the same pathogen) and combination vaccines (targeting multiple pathogens). Across the reviewed pipeline, there are 32 monovalent, nine bivalent, and 46 multivalent (three or more strains) candidates. Strain valency data were unavailable for four candidates (Figure 9).

### 3.4. Pipeline Evolution over Time (2022–2025)

This section summarises year-to-year changes in the VE clinical-stage pipeline between 2022 and 2025 [29,34,35,36], using the longitudinal indicators defined in Section 2.3 (i.e., attrition, registration, progression, and entry rates), alongside descriptive trends in pipeline composition.

- Overall pipeline size and composition

Across the period, the total number of candidates was 100 in 2022, 103 in 2023, 98 in 2024, and 91 in 2025, while VE membership varied with 15 in 2022, 16 in 2023, 14 in 2024, and 16 in 2025.

Each year, the vast majority (95%) of the pipeline focused on prophylactic approaches, with the remaining 5% designed for therapeutic use. All stages of clinical development were represented throughout the period, although the share of late-stage candidates (Phase III and regulatory review) decreased from 42% in 2022 to 31% in 2023 and 22% in 2024, before rising to 25% in 2025.

Across 2022–2025, the pipeline remained consistently composed of approximately 40–45% of candidates targeting diseases, disease combinations, or infectious syndromes for which no vaccine or prophylactic mAb had been registered globally. The remaining candidates represented further development of existing interventions, as defined in the previous section.

b. Pipeline flow dynamics

**Figure 10:**
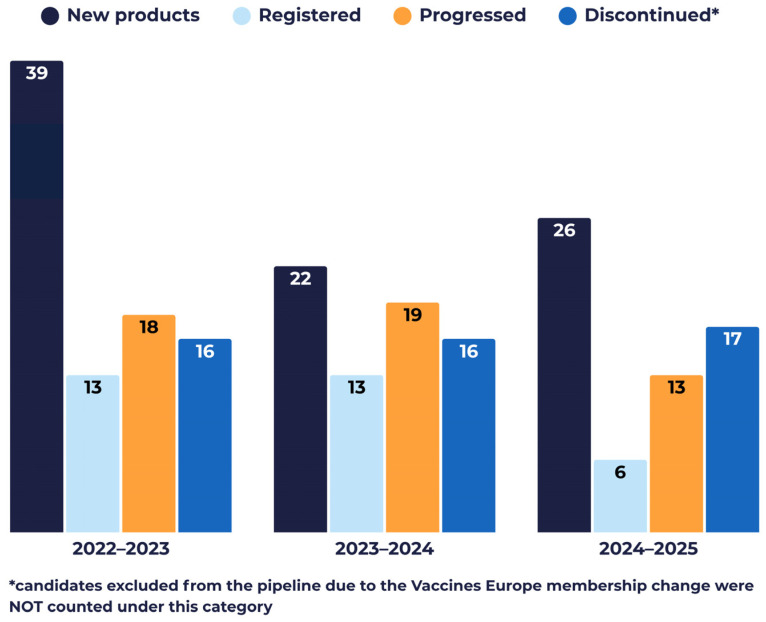
Evolution of the pipelines of Vaccines Europe member companies between 2022 and 2025.

Since 2022, the pipeline has been actively reshaped through candidate discontinuations, regulatory approvals, progression across development phases, and the entry of new candidates. Cumulatively, 49 development programmes were discontinued (note that this does not include candidates from companies no longer members of VE and therefore no longer accounted for in the pipeline), 32 candidates received marketing authorisation, 50 candidates progressed through clinical development phases, and 87 new candidates entered the pipeline (Figure 10). Year-to-year changes were assessed using four annual indicators, calculated as described in Section 2.3 (Table 1):

- The annual attrition rate remained relatively stable across the period at approximately 16% (range: 15.5–17.3%).
- Over the same timeframe, the annual registration rate averaged approximately 11% (range: 6.1–13.0%), reflecting the proportion of candidates receiving marketing authorisation each year.
- Progression across clinical development phases also remained consistent, with an average of 17% (range: 13.3–18.4%) of the pipeline advancing annually, indicating continued movement of candidates through the pipeline despite discontinuations.
- Pipeline entry was highest in the 2022–2023 interval, with 39% (39) new candidates entering clinical development, possibly reflecting accelerated activity in the context of the COVID-19 pandemic. In subsequent years, entry stabilised, with approximately 21% (22) of candidates entering the pipeline in 2023–2024 and 27% (26) in 2024–2025.

Despite the caveat that VE membership changes over the years may impact numbers, these indicators collectively point to a sustained pipeline turnover, characterised by steady attrition and progression, ongoing regulatory output, and continued entry of new clinical-stage candidates (Figure 10).

c. Shifts in portfolio focus over time

Between 2022 and 2025, most candidates (approximately 75% over the period) targeted infectious diseases caused by viruses. A smaller but stable proportion (approximately 23% over the period) targeted bacterial infections, and only 2% targeted protozoal infections in 2022–2024, with none represented in 2025. In 2022, 5% of the candidates had no disclosed target microorganism.

During the same period, most candidates targeted adults, representing the largest share throughout the period (35–40% range, averaging 38%). A consistent but smaller proportion focused exclusively on paediatric populations, remaining relatively stable (14–18% range, averaging 15%). Programmes targeting both adults and older adults increased steadily over time, rising from 19% in 2022 to 30% in 2025, indicating a growing emphasis on broader adult age coverage.

Sexually transmitted infections remained a small part of the portfolio, but the number of candidates has been increasing from four candidates in 2023 to six in 2025 (around 7% of the 2025 pipeline). These programmes included vaccines targeting chlamydia, HSV, HIV, HPV, and mpox. Infection-associated cancer prevention was also present in the pipeline, with four prophylactic candidates in 2025 (three targeting EBV; one HPV).

Respiratory pathogens remained the dominant focus throughout the period, accounting for approximately 75–80% of candidates. Candidates targeting SARS-CoV-2, alone or in combination, or other coronaviruses, decreased as a share of the pipeline from 30% in 2022 to 14% in 2025. Conversely, candidates targeting seasonal or pandemic influenza increased from 12% in 2022 to 33% in 2025.

In proportional terms, AMR-relevant candidates represented 11% of the total pipeline in 2022, 15% in 2023, 14% in 2024, and reaching 19% in 2025. This suggests an upward trend in the relative importance of AMR-focused development over the 2022–2025 period.

Between 2022 and 2025, the number of candidates targeting vector-borne diseases showed a gradual decline, decreasing from ten in 2022 and 2023 to eight in 2024 and seven in 2025, although it is to be noted that two vector-borne disease vaccines were registered over this period. In contrast, candidates targeting food-borne and water-borne diseases remained very limited in 2022 and 2023 (one candidate each year), but increased substantially in 2024 and 2025, reaching eight candidates in 2024 and six in 2025.

d. Technology trends

The pipeline continued to reflect a broadening technology base. Candidates using RNA-based and subunit approaches represented approximately 80% of the pipeline on average across 2022–2025 (78% (78) in 2022, 75% (77) in 2023, 78% (76) in 2024, and 81% (74) in 2025). Over the period, the share of RNA-based candidates increased (from 37% (37) in 2022, 42% (43) in 2023, and 48% (47) in 2024 to 53% (48) in 2025), while the share of subunit approaches (including protein, toxoid, glycoconjugate, and virus-like particle strategies) decreased (from 41% (41) in 2022, 33% (34) in 2023, and 30% (29) in 2024 to 29% (26) in 2025). Emerging approaches such as GMMA, MAPS, and prophylactic monoclonal antibodies appeared from 2023 onwards and accounted for approximately 5% of candidates in 2025, which is comparable to the share represented by live-attenuated and whole-inactivated vaccines.

## 4. Discussion

### 4.1. Interpretation of Pipeline Trends

The composition of a clinical-stage pipeline can provide an important signal of how vaccine innovation priorities align with evolving public health needs. While pipeline data do not predict licensure outcomes, they offer a structured view of where current development efforts are concentrated and where gaps or unmet needs may persist.

Current trends in VE members’ pipelines indicate a progressive shift toward approaches that enhance prevention, preparedness, and health system resilience. While 59% of candidates focus on further developing existing vaccines by improving formulation, expanding the target population, or increasing valency, an important 41% (n = 37) are directed at disease targets with no registered preventive biologic. This reflects a mix of development activity, including both novel targets in priority areas where advances in vaccine development have the potential to deliver meaningful impact (e.g., endemic diseases, infections that can lead to cancers, and therapeutic approaches against infectious agents), and approaches building on existing preventive solutions (e.g., novel combination vaccines). This balance highlights both incremental advances and a robust effort to open new prevention frontiers, offering major public health opportunities but also underscoring the scientific and regulatory challenges inherent to first-in-class development.

Protecting people at all stages of life through life-course immunisation has emerged as a central feature of the current pipeline. In 2025, 85% of the candidates in the VE members’ pipeline were being evaluated in adults and/or older adults, reflecting a shift from childhood-focused immunisation toward broader lifespan prevention. This distribution aligns with demographic trends in Europe and the growing burden of severe infectious disease in ageing populations.

Respiratory-transmitted pathogens consistently account for the largest share of candidates in the pipeline. This reflects their substantial and recurrent public health burden, as well as the experience of the COVID-19 pandemic, which underscored both vulnerability to outbreaks and the importance of scalable vaccine platforms. Beyond individual pathogens, the pipeline also signals a shift toward syndromic and combination approaches, such as, for example, vaccines targeting multiple respiratory agents. Such strategies may enhance programmatic efficiency, improve uptake, and support integrated prevention efforts, particularly in older adults and other high-risk groups. Continued development of next-generation and potentially universal influenza vaccines further illustrates a preparedness-oriented approach, aiming to address antigenic drift and pandemic potential.

AMR is widely recognised as a major global health threat, with drug-resistant infections associated with longer hospital stays, higher healthcare costs, and increased mortality [37]. In this context, the presence in the pipeline of multiple candidates targeting bacterial pathogens identified by the WHO as priorities [26], for AMR is notable, given the scientific and commercial challenges associated with bacterial vaccine development, such as antigenic variability, complex correlates of protection, and the need to demonstrate impact beyond direct disease prevention. Vaccination can support AMR mitigation by preventing infections and reducing antibiotic exposure at the population level, both by lowering the burden of bacterial disease and by reducing antibiotic prescribing linked to viral respiratory infections and secondary bacterial complications [15]. Translating this pipeline activity into measurable AMR impact will depend on sustained investment, appropriate incentive mechanisms, and value assessment frameworks that capture outcomes beyond immediate clinical endpoints, including avoided antibiotic use and reduced transmission of resistant strains.

A subset of candidates in the pipeline target vector-borne, zoonotic, or environmentally mediated pathogens whose transmission dynamics are influenced by climate conditions, ecosystem disruption, and cross-species spillover. These pathogens include agents with epidemic or pandemic potential, as well as diseases whose geographic distribution may shift as environmental conditions change. While several, such as dengue or chikungunya, have historically been associated with tropical or subtropical regions, they are increasingly relevant to Europe as reflected by reports from the European Centre for Disease Prevention and Control (ECDC) [38], which recently strengthened surveillance of vector-borne diseases. Robust surveillance and early warning systems are essential to inform timely public health decision-making and to support the adaptation of national immunisation programmes should such diseases become established or endemic in new settings. The development of candidates targeting these pathogens indicates increasing alignment between vaccine research priorities and evolving epidemiological risks in Europe and globally.

The VE members’ clinical pipeline is characterised by a large number of vaccine technologies, with 12 distinct approaches represented in 2025. At the same time, over half of the candidates are based on RNA approaches, reflecting sustained development activity and the continued use of platform technologies that were rapidly advanced during the COVID-19 pandemic. The presence of a broad mix of established and emerging approaches suggests that innovation is progressing along multiple scientific pathways, supporting efforts to address pathogens with different biological characteristics and to meet diverse population needs. In parallel, the pipeline shows a strong emphasis on broadened antigen coverage through multivalent approaches, which represent approximately 60% of vaccine candidates in 2025. This trend reflects ongoing efforts to strengthen protection in the context of pathogen diversity, geographic variation, and antigenic evolution, particularly for diseases where multiple strains or serotypes circulate or where immune escape is an ongoing concern.

Finally, the pipeline includes candidates reflecting mechanism-driven and preparedness-oriented innovation. Examples include Lyme disease vaccines targeting outer surface protein A (OspA) [28], which aim to interrupt Borrelia transmission at the vector level before human infection. Similarly, RSV vaccines and extended half-life monoclonal antibodies are broadening protection for both infants [2] and older adults, while universal influenza vaccine candidates aim to achieve cross-strain immunity to mitigate seasonal and pandemic threats. Ongoing advances in tuberculosis vaccines, next-generation COVID-19 vaccines, and candidates targeting antimicrobial-resistant pathogens further highlight an increasing focus on complex infectious disease challenges. Collectively, these developments illustrate a transition toward integrated and forward-looking immunisation strategies designed to strengthen long-term public health preparedness.

### 4.2. Limitations of the Analysis

This descriptive analysis has several limitations related to its scope, the data selection criteria, and the nature of the data collection methodology.

First, its scope is limited to publicly available information on infectious disease vaccines and prophylactic mAbs in clinical development reported by VE member companies as of the data collection cut-off (end of August of the given year). Since the dataset is based on publicly available information supplemented by validation from participating companies, there is a potential for missing information, classification, and interpretative bias. While predefined classification rules were applied consistently, the analysis reflects aggregated industry-reported data and may be subject to inherent limitations in source reporting and interpretation. Preclinical development was excluded, as early-stage programmes are often not publicly disclosed for strategic and competitive reasons.

Second, the analysis reflects the activities of VE member companies only and, as such, does not provide an exhaustive view of all clinical-stage infectious disease vaccine and prophylactic mAbs developed globally. VE membership has evolved over time, from 15 companies in 2022 to 16 in 2023, 14 in 2024, and 16 in 2025, which could impact pipeline trends over those years. Despite those changes, a subset of 10 companies remained members across the 2022–2025 period considered for the longitudinal analysis part of this descriptive analysis. These constant members accounted for 90 to 96% of all candidates each year, suggesting that the principal longitudinal patterns are unlikely to be driven solely by membership turnover. Nevertheless, because the analysis is based on aggregated annual datasets rather than a fixed company-level cohort analysis, year-to-year comparisons should be interpreted with caution.

Finally, the review focuses on infectious disease prevention and does not capture candidates targeting non-infectious indications, including therapeutic vaccines for cancer or vaccines and immunotherapies under development for rare diseases.

Despite these limitations and the fact that clinical development of a candidate, even in an advanced stage, does not necessarily guarantee the success of the programme or eventual availability of a licensed vaccine, this descriptive analysis provides a yearly snapshot of vaccine innovation within the industry.

### 4.3. Drivers and Constraints Shaping the Pipeline

The global vaccine research environment continuously evolves in response to a changing health landscape, with cutting-edge science driving the development of innovative immunisation solutions. This adaptability is key to addressing a diversity of pathogens and broadening the range of available immunisation tools, allowing prevention strategies to better match population needs across settings.

Vaccine development is shaped by multiple factors beyond clinical study outcomes. High attrition remains an inherent feature of vaccine development, and inclusion of a candidate in the clinical pipeline does not imply eventual licensure. Vaccine development is typically long and resource-intensive: timelines commonly span a decade or more from early development to approval, reflecting the complexity of generating robust safety, immunogenicity, and efficacy evidence across populations. Published cost estimates vary widely depending on assumptions and failure rates, with clinical development accounting for a substantial share of total R&D investment [39]. Scientific complexity remains a key driver of feasibility and attrition, including the need to bridge gaps in antigen and epitope discovery, improve understanding of pathogen structure and pathogen–host interactions, and identify immune mechanisms and correlates of protection that can guide candidate selection. These challenges are particularly relevant for pathogens characterised by antigenic variability or complex host–pathogen dynamics.

A further scientific challenge lies at the interface between preclinical and clinical phases. Evidence-based selection of candidates for clinical trials relies on preclinical models that do not always predict human immune responses. Bridging the knowledge gap between mechanisms of immunisation in experimental systems and in clinical trial participants is therefore critical to improve translatability and reduce attrition. Advances in human challenge studies, developments in New Approach Methodologies (NAMs), improved biomarker discovery and validation, including the identification of correlates of protection, and the application of computational and data-driven modelling approaches, including machine learning-enabled analyses, are increasingly important tools to strengthen candidate selection and optimise trial design.

Operational and epidemiological realities also influence pipeline composition. Vaccine clinical trials often require large populations, extended follow-up periods, and complex multi-site coordination. These challenges are amplified for pathogens with seasonal transmission, geographically variable incidence, or outbreak-driven epidemiology, where trial feasibility may depend on rapidly changing local contexts. Clinical development is further affected by regulatory, bureaucratic, and logistical complexity in multinational studies, resource constraints (including funding, research infrastructure, and trained personnel), and the need to adapt to shifting pathogen circulation patterns and antigenic evolution. Beyond clinical trial execution, vaccine development and deployment pathways must also account for practical requirements linked to scale-up and supply, including manufacturing scalability, quality assurance systems aligned with regulatory standards, and distribution logistics, and an innovation-enabling policy framework that supports and incentivises research and development, all of which can influence development decisions and timelines.

### 4.4. Considerations for Policy and Public Health Strategy

The following considerations provide broader context and interpretative perspectives on the implications of these findings and are not derived directly from the descriptive analysis presented.

Translating a diverse clinical pipeline into public health impact depends on the strength of the broader innovation and access environment. A 2024 IQVIA report mandated by EFPIA/VE identified that the share of global immunisation clinical trials conducted in Europe fell from 17% in 2018 to 8% in 2023 while it increased in Asia over the same period (i.e., China 14% to 21% and Japan 6% to 9%) [40], suggesting a declining attractiveness of the region for developers and trial sponsors. This highlights the importance of multi-year, predictable investment, expanded research infrastructure and workforce capacity, alongside streamlined and more predictable regulatory and clinical trial processes (e.g., simplified multi-country trials, reduced administrative burden) to sustain innovation, de-risk development, and maintain Europe’s competitiveness in vaccine R&D.

Robust surveillance and evidence-generation capabilities are essential to inform development choices and support lifecycle assessment of value. However, persistent challenges in timely and standardised data reporting across national and EU levels can limit the generation of reliable real-world evidence and delay assessment of vaccine performance and value. Addressing these gaps through greater digitalisation, interoperable information systems, and high-quality immunisation registries will be important to support both research and policy.

Fragmentation across Member States in areas such as clinical trial approvals, regulatory authorisation, health technology assessment (HTA), and procurement can result in divergent evidence requirements, heterogeneous funding decisions, and unpredictable access pathways, particularly for innovative vaccines targeting new indications or populations. Greater EU-level coordination of scientific development and regulatory approaches, together with more coherent and predictable HTA processes, including expert NITAGs (National Immunisation Technical Advisory Groups) involvement in the EU Joint Clinical Assessment (JCA) process for vaccines from 2030, could support more predictable pathways, accelerate access, and improve equity across the region, while respecting national competencies. In parallel, agreed evidentiary standards and enhanced real-world data generation can support lifecycle value demonstration, post-authorisation evidence needs, and more consistent HTA outcomes. Improved procurement practices at the national level, including the use of multi-year, value-based and resilience-oriented approaches, and tender designs that avoid a sole focus on the lowest price and recognise the broader value and security of supply of vaccines, could further reduce uncertainty for manufacturers and support sustainable supply.

The pipeline also reinforces the importance of assessment and incentive frameworks that reflect the broader societal value of vaccines, including life-course protection, health system resilience (including in the context of climate change), AMR mitigation, and preparedness for outbreaks and pandemics. Equally important is attention to vaccine acceptance, as public confidence can influence uptake across immunisation programmes. Integrating effective risk communication, community engagement, and transparent information about safety and regulatory processes into deployment strategies will be essential to maximise uptake and public health impact. Such considerations may be particularly relevant for socially valuable yet commercially challenging development areas, including bacterial vaccines relevant to AMR. Incentive approaches may include a mix of pull and push mechanisms, complemented by regulatory guidance and HTA frameworks able to account for outcomes such as avoided infections, reduced antibiotic use, and prevented resistance. Aligning these frameworks with the EU’s broader competitiveness and innovation agenda, including initiatives such as the EU Biotech and industrial strategies, will be important to ensure that Europe remains an attractive location for vaccine R&D, manufacturing, and deployment.

Finally, the pipeline’s strong orientation toward adult and older adult populations underscores the need to institutionalise life-course immunisation through aligned and sustainable financing, access, and delivery systems. In particular, strengthening vaccination infrastructure, including efficient delivery systems, a trained healthcare workforce across care settings, and effective digital tools (e.g., for scheduling, reminder–recall, and coverage monitoring) will be critical to achieving high and equitable uptake of vaccines across the life-course.

## 5. Conclusions

The VE 2025 Pipeline Review provides a structured, annual overview of clinical-stage vaccines and prophylactic monoclonal antibodies targeting infectious diseases and their consequences being developed by VE member companies. As the first initiative of its kind to consolidate and transparently track European industry clinical pipeline activity using a consistent methodology, it offers a forward-looking signal of innovation priorities and emerging areas of focus. The 2025 pipeline is characterised by a predominance of candidates targeting respiratory-transmitted pathogens, increasing emphasis on adult and older adult populations, sustained activity in bacterial pathogens relevant to antimicrobial resistance, and continued diversification of vaccine technologies. The inclusion of candidates addressing climate-sensitive and zoonotic threats further reflects alignment with evolving epidemiological risks.

Collectively, these trends suggest that vaccine development is increasingly shaped by preparedness considerations and long-term prevention strategies across the life course. By providing a recurring, comparable snapshot of clinical-stage innovation, this review highlights both the breadth of ongoing development activity and the scientific and translational challenges associated with complex pathogens, including bacterial threats and diseases with shifting epidemiology.

VE’s role as a convenor and catalyst for this effort supports regular horizon scanning and evidence-based dialogue between the vaccine industry and health authorities. Continuous exchange between developers and public health institutions, including the European Centre for Disease Prevention and Control (ECDC) and Health Authorities, is essential to ensure alignment between evolving epidemiological trends, surveillance data, and vaccine development priorities. Sustaining and translating innovation into public health impact will require predictable investment, robust surveillance and evidence generation, and policy environments that support timely access and uptake. Continued systematic monitoring of pipeline trends can help strengthen strategic preparedness planning and reinforce immunisation as a core component of Europe’s long-term health security.

## Figures and Tables

**Table 1:** Pipeline changes over 2022–2025 period.

	Previous Year Pipeline Denominator	Discontinued	Registered	Progressed	New	Net Change	Attrition Rate	Registration Rate	Progression Rate	Entry Rate
**2022–2023**	100	16	13	18	39	10	16.0%	13.0%	18.0%	39.0%
**2023–2024**	103	16	13	19	22	−7	15.5%	12.6%	18.4%	21.4%
**2024–2025**	98	17	6	13	26	3	17.3%	6.1%	13.3%	26.5%
**Total**	187	49	32	50	87	6	N/A *	N/A	N/A	N/A
**Average rates**	N/A	N/A	N/A	N/A	N/A	N/A	16.3%	10.6%	16.6%	29.0%

* Non-applicable.

## Data Availability

The data analysed in this study were derived from publicly available sources cited in the manuscript, including the Vaccines Europe Pipeline Reviews and company public pipeline information. Aggregated data supporting the findings are presented within the article and in Annex of the cited Vaccine Europe Pipeline Reviews.

## Abbreviations

The following abbreviations are used in this manuscript:

AMR	Antimicrobial resistance
CMV	Cytomegalovirus
COVID-19	Coronavirus disease of 2019
EBV	Epstein–Barr virus
ECDC	European Centre for Disease Prevention and Control
EFPIA	European Federation of Pharmaceutical Industries and Associations
EU	European Union
GAVI	Global Alliance for Vaccines and Immunisation
GBS	Group B *Streptococcus*
GMMA	Generalised Modules for Membrane Antigens
HERA	Health Emergency Preparedness and Response Authority
HIV	Human immunodeficiency virus
HPV	Human papillomavirus
HSV	Herpes simplex virus
HTA	Health technology assessment
hMPV	Human metapneumovirus
JCA	Joint clinical assessment
MAPS	Multiple antigen-presenting systems
MMRV	Measles, mumps, rubella, and varicella
NAMs	New approach methodologies
NITAG	National Immunisation Technical Advisory Group
mAbs	Monoclonal antibodies
OspA	Outer surface protein A
PIV3	Parainfluenza type 3
R&D	Research and development
RNA	Ribonucleic acid
RSV	Respiratory Syncytial Virus
UPEC	Uropathogenic *Escherichia coli*
VE	Vaccines Europe
VLPs	Virus-like particles
WHO	World Health Organisation
